# Do Cryptic Reservoirs Threaten Gambiense-Sleeping Sickness Elimination?

**DOI:** 10.1016/j.pt.2017.11.008

**Published:** 2018-03

**Authors:** Informal Expert Group on Gambiense HAT Reservoirs, Philippe Büscher, Jean-Mathieu Bart, Marleen Boelaert, Bruno Bucheton, Giuliano Cecchi, Nakul Chitnis, David Courtin, Luisa M. Figueiredo, José-Ramon Franco, Pascal Grébaut, Epco Hasker, Hamidou Ilboudo, Vincent Jamonneau, Mathurin Koffi, Veerle Lejon, Annette MacLeod, Justin Masumu, Enock Matovu, Raffaele Mattioli, Harry Noyes, Albert Picado, Kat S. Rock, Brice Rotureau, Gustave Simo, Sophie Thévenon, Sandra Trindade, Philippe Truc, Nick Van Reet

**Affiliations:** 1 Department of Biomedical Sciences, Institute of Tropical Medicine, Nationalestraat 155, 2000 Antwerp, Belgium; 2 INTERTRYP, IRD, CIRAD, Univ Montpellier, Montpellier, France; 3 Centro Nacional de Medicina Tropical, Instituto de Salud Carlos III, Calle Sinesio Delgado 4, 28029 Madrid, Spain; 4 Department of Public Health, Institute of Tropical Medicine, Nationalestraat 155, 2000 Antwerp, Belgium; 5 Sub-regional Office for Eastern Africa, Food and Agriculture Organization of the United Nations, CMC Road, Bole Sub City, Kebele 12/13, P O Box 5536, Addis Ababa, Ethiopia; 6 Department of Epidemiology and Public Health, Swiss Tropical and Public Health Institute, Socinstrasse 57, Postfach, 4002 Basel, Switzerland; 7 University of Basel, Switzerland; 8 Université Paris Descartes, Institut de Recherche pour le Développement, Unité MERIT, Mère et enfant face aux infections tropicales, 4 avenue de l’Observatoire, 75006 Paris, France; 9 Instituto de Medicina Molecular, Faculdade de Medicina, Universidade de Lisboa, Avenida Prof Egas Moniz, 1649-028 Lisboa, Portugal; 10 Control of Neglected Tropical Diseases, Innovative and Intensified Disease Management, World Health Organization, Via Appia 20, 1202 Geneva, Switzerland; 11 Institut de Recherche sur les Bases Biologiques de la Lutte Intégrée, Centre International de Recherche-Développement sur l’Élevage en zone Subhumide, 01 BP 454 Bobo-Dioulasso 01, Burkina Faso; 12 Université Jean Lorougnon Guédé, BP 150 Daloa, Côte d’Ivoire; 13 Institute of Biodiversity, Animal Health and Comparative Medicine, University of Glasgow, Henry Wellcome Building, 464 Bearsden Road, Glasgow, UK; 14 Département de Parasitologie, Institut National de Recherche Biomédicale, Avenue de la Démocratie, BP 1197 Kinshasa 1, République Démocratique du Congo; 15 College of Veterinary Medicine, Animal Resources and Biosecurity, Makerere University, P O Box 7062 Kampala, Uganda; 16 Animal Production and Health Division, Food and Agriculture Organization of the United Nations, Viale delle Terme di Caracalla, 00153 Rome, Italy; 17 Institute of Integrative Biology, University of Liverpool, Liverpool L69 7ZB, UK; 18 Foundation for Innovative New Diagnostics, 9 Chemin des Mines, 1202 Geneva, Switzerland; 19 Zeeman Institute for Systems Biology & Infectious Disease Research, University of Warwick, Gibbet Hill Road, Coventry CV4 7AL, UK; 20 Trypanosome Transmission Group, Trypanosome Cell Biology Unit, INSERM U1201 and Department of Parasites and Insect Vectors, Institut Pasteur, 25, rue du Docteur Roux, 75015 Paris, France; 21 Department of Biochemistry, Faculty of Science, University of Dschang, P O Box 67 Dschang, Cameroon; 22 CIRAD, INTERTRYP, Montpellier, France

**Keywords:** human African trypanosomiasis, *Trypanosoma brucei gambiense*, reservoir, sleeping sickness, transmission, elimination

## Abstract

*Trypanosoma brucei gambiense* causes human African trypanosomiasis (HAT). Between 1990 and 2015, almost 440 000 cases were reported. Large-scale screening of populations at risk, drug donations, and efforts by national and international stakeholders have brought the epidemic under control with <2200 cases in 2016. The World Health Organization (WHO) has set the goals of *gambiense*-HAT elimination as a public health problem for 2020, and of interruption of transmission to humans for 2030. Latent human infections and possible animal reservoirs may challenge these goals. It remains largely unknown whether, and to what extend, they have an impact on *gambiense*-HAT transmission. We argue that a better understanding of the contribution of human and putative animal reservoirs to *gambiense-*HAT epidemiology is mandatory to inform elimination strategies.

## Highlights

*gambiense*-HAT is targeted for elimination with zero transmission in humans.

Innovative tools may contribute to the achievement of elimination; these tools include rapid diagnostic tests, improved tsetse-control tools, and an oral drug to treat both stages of disease.

Research is revealing associations between infection outcome, including self-cure, and mutations within genes involved in immune responses.

Patient-derived *T. b. gambiense* strains can cycle in animals and tsetse flies without losing infectivity to humans. Molecular and serological techniques facilitate new studies on naturally infected animals as putative reservoir hosts.

Mathematical modelling supports the hypothesis that human or animal reservoirs drive transmission, and they, or the tsetse vectors, could be targeted to swiftly impact transmission. Ongoing modelling will assess possible recrudescence via reservoirs.

## Can Cryptic Reservoirs in Humans and Animals Compromise the Sustainable Elimination of *gambiense*-HAT?

HAT is caused by two closely related parasites that are transmitted by tsetse flies. *Trypanosoma brucei gambiense* is responsible for the Western and Central African form of the disease and *Trypanosoma brucei rhodesiense* occurs in Eastern and Southern Africa – both forms of the disease are usually fatal if untreated [1]. Between 1990 and 2016, a total of 437 971 cases of *gambiense*-HAT were reported, with a peak of 37 385 cases in 1998i. Thanks to large-scale deployment of a serological screening test (**CATT/*T. b. gambiense***) (see Glossary), drug donations, and intense efforts by national and international stakeholders, this epidemic has been brought under control, with fewer than 2200 cases reported in 2016. This represents a marked reduction in human suffering caused by the disease. Inspired by this progress, the WHO has set **elimination** of *gambiense*-HAT as a target for the near future: **elimination as a public health problem** by 2020 and the interruption of transmission to humans by 2030ii.

The rationale to shift from HAT control to elimination is based on several arguments, such as the epidemiological vulnerability of *gambiense*-HAT as a presumed **anthroponotic** infection, historic examples of elimination in several West African foci, the availability of new medicines and diagnostics, the political will of endemic countries, and the commitment of national control programs [2]. Furthermore, a drug donation agreement between pharmaceutical companies and WHO has made treatment freely available to endemic countries.

*gambiense*-HAT control classically relies on three pillars: vector control, case finding, and treatment. HAT is a vector-borne disease, and the reduction of human–fly contact below a critical threshold would lead to zero transmission. Although vector control is critical to achieve the elimination/eradication goals, in practice, it will be hard to sustain control of all tsetse fly populations in all endemic countries. Vector control being only part of the solution, *gambiense*-HAT control will continue to rely to a great extent on surveillance, diagnosis, and treatment, both for reducing transmission and for monitoring progress towards these goals.

The introduction of individual **rapid diagnostic tests** (RDTs) for *gambiense*-HAT may increase serological screening coverage as they can be performed in remote dispensaries devoid of technical facilities. Thus, they facilitate the integration of passive screening in the health system and play a role in a sustainable surveillance system. However, RDTs also have limitations – like CATT/*T. b. gambiense*, they only detect antibodies, and their **specificity** is not 100% [3]. As a consequence, given the adverse effects and logistic constraints of current treatment, individuals who test positive in an RDT or in CATT must undergo microscopic examination of blood or lymph node fluid to confirm the presence of the parasite, followed by a lumbar puncture for **stage determination,** as different drugs are required to treat early- and late-stage disease [2]. In recent years, the highly toxic melarsoprol regimen, used to treat late-stage disease, has been replaced by a safer, though still rather complex, treatment requiring parenteral administration and hospitalisation. An oral treatment might become available in late 2018, and a single-dose treatment is entering phase III clinical trialsiii [4].

Whereas HAT elimination by 2020, as a public health problem, seems within reach, the sustained global elimination of HAT appears more challenging. Indeed, as long as the knowledge gaps surrounding the **reservoir** of *T. b. gambiense* in interepidemic periods are not filled, the concept of **eradication** of *gambiense*-HAT cannot be considered.

We present the current research evidence about potential human and animal *T. b. gambiense* reservoirs and discuss their importance in the light of the *gambiense*-HAT elimination goals.

## Human Reservoir

Mathematical models show that the sustained transmission of HAT can be explained if a fraction of the HAT cases is systematically missed by the screening operations [5]. Unfortunately, this is the case in many settings as a number of *T. b. gambiense* infections remain undiagnosed for several reasons [6]. First, not all infected people are reached by screening activities. Second, current diagnostic techniques do not pick up all *T. b. gambiense* infections due to lack of sensitivity of serological screening tests, of molecular techniques, or of the parasitological confirmation tests [7]. These undiagnosed, yet infected, people will act as a human reservoir of the parasite and might sustain transmission, forming a **maintenance population** [8]. Still another potential category of human reservoir may consist of **latent infections**, also called ‘healthy carriers’, who do not always progress to clinical disease, though the relative contribution of these individuals to parasite transmission still needs to be documented (Box 1). These latently infected people may carry trypanosomes for years or even decades, as was first described half a century ago in West Africa, and later in patients refusing treatment in Côte d’Ivoire 9, 10. More recently, a HAT case with a latent infection of at least 29 years was documented [11]. Whether latently infected persons transmit the parasite sexually [12], and whether sexual and congenital transmission plays a significant role in the epidemiology of *gambiense*-HAT [13], remains hypothetical. In Guinea, asymptomatic or latent infections were found to have consistently high titres in CATT/*T. b. gambiense* and to be positive in the **immune trypanolysis** test, although no parasites could be detected in blood or lymph node fluid during a 2-year follow-up period [14]. This observation is in line with the fact that trypanosomes can survive in the extravascular spaces of diverse organs such as the heart, the central nervous system, and the skin 15, 16, 17. Experimental infections in animals confirmed that parasites may be undetectable in the blood but hidden in different organs and tissues 18, 19, 20, 21, including the skin, from where they can be ingested by tsetse flies 22, 23. It was only recently that researchers began to investigate the underlying host–parasite interaction mechanisms responsible for those latent infections. Microsatellite profiles and genomic sequencing of parasites from latent infections and from clinical HAT patients are indistinguishable, suggesting that the latent infection phenotype is determined primarily by the host rather than by the parasite [24]. Studies on host genetic polymorphism show that *tumor necrosis factor-α-308* *A*, *HLA-G UTR-2*, *APOL1 N264K*, *and APOL1 G2* are associated with increased risk of infection or with disease progression, while *IL10_-592_ A*, *IL6*_4339_, *APOL1 G1*, and other polymorphisms in *HPR and APOL1* are associated with decreased risk of infection or with latent infection 25, 26, 27, 28, 29, 30. Other studies have found associations between the innate and the adaptive immune response and infection outcome, for example, **self-cure** and high levels of interleukin-8 (IL-8); latent infection and high levels of IL-6 or specific interferon-γ-producing T cells; disease progression and high levels of IL-10, TNF-α, and sHLA-G 31, 32, 33. In view of the global elimination of HAT, it is of the utmost importance to clarify the extent to which these human reservoirs contribute to the transmission of the parasite and hence to *gambiense*-HAT persistence and potential resurgence.

**Figure I:**
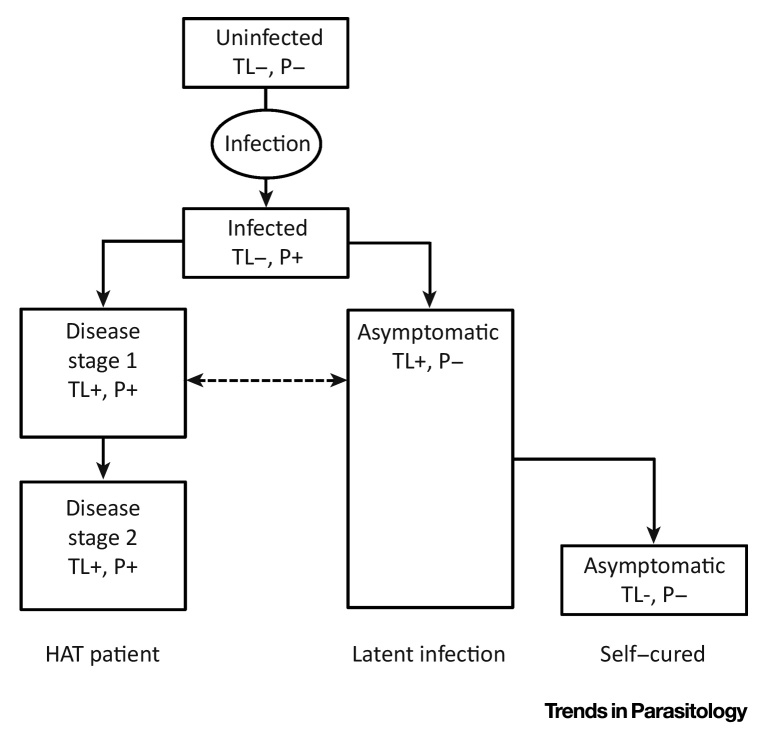
Outcomes of Human Infection with *Trypanosoma brucei gambiense*. get infected with T. When naïve persons (uninfected), without specific antibodies (TL−) and without parasites (P−) become infected with *T. b. gambiense*, they undergo an early phase of the disease with detectable parasitaemia (P+) but without detectable specific antibodies. Thereafter, most of them develop the disease (HAT patient) and are characterised by specific antibodies (TL+) and detectable parasitaemia (P+). Some remain asymptomatic (latent infection) with detectable specific antibodies but without detectable parasites (TL+, P−). Evidence for self-cure comes from asymptomatic people who also eventually become negative for specific antibodies (TL−, P−).

Box 1 **Diversity in Outcomes of Human Trypanosoma brucei gambiense Infections**

There is growing evidence that infection with *T. b. gambiense* does not always follow the classical course of the disease, that is, a first haemolymphatic stage followed by a second stage with central nervous system involvement progressing to death if left untreated (Figure I). These symptomatic HAT patients are characterised by the detection of parasites in any body fluid (P+), detection of specific antibodies against *T. b. gambiense* Variable Antigen Type LiTat 1.3 or LiTat 1.5 in immune trypanolysis (TL+), and the presence of clinical symptoms. However, long-term follow-up studies in West Africa have shown that a number of infected individuals do not develop the disease and can be classified as having latent infections (i.e., they are healthy carriers) [9]. They remain asymptomatic without detectable parasites (P−) for several years, although they are consistently positive in the immune trypanolysis test (TL+). Moreover, some of them may become immune trypanolysis-negative (TL−) over time, suggesting that they self-cured and therefore cannot transmit the parasite any more.

Alt-text: Box 1

## Animal Reservoir

**Figure 1:**
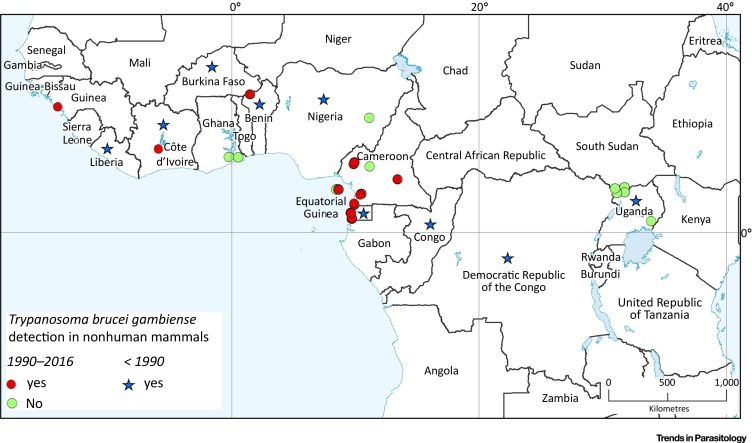
*Trypanosoma brucei gambiense* in Nonhuman Mammals. The map shows *gambiense*-human African trypanosomiasis in endemic countries and sites where *T. b. gambiense* infection in nonhuman mammals has been investigated with direct and indirect methods. Circles represent direct or indirect evidence of presence (red) and of absence (green) of *T. b. gambiense* in the period 1990–2016. For this period, data are mapped at the village/site level. (Blue) stars represent presence of detection in the years prior to 1990. For this period, data are mapped at the country level. All source references are provided in Tables S1 and S2 in the supplemental information online.

Compared to latent infections in humans, our current knowledge of *T. b. gambiense* infections in animals is very limited and fragmented. The presence of *T. b. gambiense* in animals has been demonstrated in several studies (Figure 1) 34, 35. Several authors have suggested that animals can act as a reservoir for *gambiense*-HAT 36, 37, 38, 39, 40, 41, 42, 43, 44, 45. In *rhodesiense*-HAT, sustained parasite transmission cycles exist in both livestock and wildlife, from which the parasite can spill over to humans [46]. For *T. b. gambiense*, despite early data generated on its infectivity and transmissibility in animals, the epidemiological significance of any animal reservoir is not well understood and may depend on the specific ecosystem of the **HAT focus**. Even if the parasite can be transmitted to and from animals, factors such as the proportion of blood-feeding on that species by tsetse will determine the epidemiological significance of the species to act as a maintenance population or part of a **maintenance community.** *T. b. gambiense* can infect a variety of domestic animals and wildlife, as shown in Table 1. Following infection, most of these animals remain asymptomatic and generally show low to very low parasitaemia. For instance, in pigs infected with a *T. b. gambiense* strain isolated from a human patient, only **xenodiagnosis** and blood culture succeeded in revealing an infection but conventional microscopy failed to detect parasites 47, 48, 49, 50, 51. Moreover, experimental studies have shown that human-derived *T. b. gambiense* strains that were cyclically transmitted by tsetse flies between animals for more than a year remained transmissible to humans [48].

**Table 1:** Animals Successfully Infected with *T. b. gambiense* Strains Isolated from Human Patients

Animal species	Origin of trypanosome strain^a^	Infectiveness to tsetse	Minimum observed duration of infection	Refs
Domestic animals				
Cat	Senegambia and Congo Free State	Not tested	12 days	[73]
Cattle	Nigeria	Yes	50 days	66, 74
Chicken	Unknown	Not tested	75 days	[75]
Dog	Senegambia and Congo Free State, Nigeria; Belgian Congo	Yes	109 days	36, 48, 73
Donkey	Senegambia	Not tested	14 days	[73]
Goat	Senegambia, Nigeria, Belgian Congo	Yes	13 months	48, 73, 74
Horse	Senegambia	Not tested	5 months	[73]
Pig	Côte d’Ivoire, Congo Belge, Nigeria	Yes	18 months	47, 51, 76
Sheep	Côte d’Ivoire	Not tested		[77]
Primates				
Agile mangabey (*Cercocebus galeritus agilis*)	Belgian Congo	Yes		[48]
Green monkey (*Cercopithecus callitrichus, C. aethiops tantalus*)	Congo Free State, Nigeria	Yes	3 months	36, 73
Wolf's mona monkey (*Cercopithecus wolfi*)	Congo Belge	Yes	15 days	[47]
Patas monkey (*Erythrocebus patas patas*)	Nigeria	Yes	3 months	36, 78
Rhesus macaque (*Macacus rhesus*)	Senegambia and Congo Free State	Not tested	1 month	[73]
Chimpanzee (*Pan satyrus, Pan troglodytes verus*)	Senegambia, Nigeria	Not tested	17 months	73, 78, 79
Dwarf galago (*Galagoides demidovii*)	République populaire du Congo	Not tested	28 days	[80]
Ungulates				
Bay duiker (*Cephalopus dorsalis*)	Belgian Congo	Yes	24 months	[48]
Waterbuck (*Kobus ellipsiprymnus*)	Uganda	Not tested		[50]
Reedbuck (*Redunca redunca*)	Uganda	Yes	15 months	[50]
Bushbuck (*Tragelaphus spekei*)	Uganda	Yes	22 months	[50]
Rodents				
Gambian pouched rat (*Cricetomys gambianus*)	République populaire du Congo	Yes	154 days	37, 80, 81
Thicket rat (*Thamnomys rutilans*), Jackson’s praomys (*Praomys jacksoni*), African marsh rat (*Dasymys incomtus)*, Striped grass mouse (*Lemniscomus striatus*), Rusty-nosed rat (*Cenomys hypoxanthus*), African brush-tailed porcupine (*Atherurus africanus*)	République populaire du Congo	Not tested	131 days	[80]

a For reasons of traceability, we use the name of countries and the scientific name of animals as mentioned in the original publication: Senegambia = Senegal and The Gambia; Belgian Congo, Congo Free State and Congo Belge = Democratic Republic of the Congo; République populaire du Congo = Republic of the Congo.

Studying natural *T. b. gambiense* infections in animals is challenging. Major drawbacks are the usually low parasitaemia and the necessity to distinguish *T. b. gambiense* from other trypanosome species such as *T. brucei brucei, T. congolense*, *T. vivax*, *T. suis*, and *T. simiae*. In particular, *T. b. gambiense* is morphologically identical to the nonhuman infective *T. b. brucei.* Among the molecular tests, only those targeting the single-copy TgsGP gene are *gambiense*-specific, thus limiting their analytical sensitivity to >100 trypanosomes per ml of blood 52, 53. Biochemical assays, such as isoenzyme profiling, are only applicable on parasite strains that have been isolated and adapted to laboratory rodents or to *in vitro* cultures 54, 55, 56, and phenotypic assays such as the **blood incubation infectivity test** are only readily applicable on isolated strains and are not fully *gambiense*-specific [57]. Tests that detect antibodies against *gambiense*-specific antigens, such as the **variant surface glycoproteins** (VSGs) LiTat 1.3 and LiTat 1.5, may be more useful in revealing *T. b. gambiense* infections in animals. However, the immune trypanolysis test (TL), which is considered 100% specific in humans, still has to be validated in different species of animals. Ancillary information on the *T. b. gambiense* animal reservoir can be drawn from analysing *T. b. gambiense* infection in tsetse, in combination with its feeding behaviour, to assess the vectorial transmission of the parasite from the animal reservoir to humans [58]. In summary, there is a need to further improve our tools and increase our understanding regarding the importance of an animal reservoir in *gambiense*-HAT epidemiology. If further evidence indicates that an animal reservoir may threaten *gambiense*-HAT elimination, synergy with the control of animal African trypanosomiasis should be considered [59].

## Filling the Knowledge Gaps

The presence of multiple reservoirs is a critical obstacle to the sustained elimination of any infectious agent [60]. For example, when the Guinea worm eradication programme was rolled out, the possibility of an animal reservoir was initially overlooked, but the recent finding of Guinea worm infections in dogs led to the hypothesis that dogs could have acted as a reservoir that caused the reappearance of human cases in Chad [61]. The existence of a human reservoir, in the form of post-kala-azar dermal leishmaniasis, and possibly also latent infections, is a challenge for the sustained elimination of visceral leishmaniasis (VL) from the Indian subcontinent [62].

The importance of investigating how HAT can re-emerge in so-called silent foci is clearly illustrated by the fact that a 9-year-old child was diagnosed with *gambiense*-HAT in Ghana in 2013, 10 years after the last detected case [63]. Also, the finding of a *gambiense*-specific PCR-positive squirrel in Equatorial Guinea on Luba island in 2014, where the last human HAT case was reported in 1995, is worrying [43]. Therefore, in the context of *gambiense*-HAT elimination, a key question is whether human and/or animal reservoirs are capable of maintaining transmission and causing a resurgence of the disease in different geographical areas and epidemiological settings (see Outstanding Questions).

As with the mathematical modelling of other neglected tropical diseases [64], models on HAT epidemiology may help to improve our epidemiological knowledge and inform elimination strategies. Models can explore if, and how, animal and human reservoirs could sustain endemicity in HAT foci [65]. However, model predictions heavily depend on the availability of accurate information for their construction, parameterisation, and fitting. To date, a few models have attempted to infer the contribution of reservoirs in *gambiense*-HAT transmission maintenance by fitting to human epidemiological data. Funk *et al*. [66] suggested that animals were necessary for persistent transmission in Bipindi focus in Cameroon. Studies of existing *gambiense*-HAT models in a few foci (i.e., D. R. Congo, Guinea, and Chad) suggest that some type of additional infection reservoir is needed to match the observed dynamics of reported HAT cases 5, 67, 68. This could arise from another human reservoir (including undiagnosed and latent infections), an animal reservoir, and/or heterogeneities in human risk exposure and surveillance coverage. A different modelling exercise considered the implications on transmission and control of whether animals function as reservoirs or as zooprophylaxis but did not address which was more likely [69].

Due to the current lack of knowledge surrounding latently infected people (including their frequency, disease progression, their relative infectivity to tsetse, and the duration of this infectious stage) modelling latent infections in humans is challenging, and these uncertainties will impact the models’ predictions. In particular, latent infections have only been explicitly incorporated in one *gambiense*-HAT model, and the potential role of these individuals in maintaining transmission or hindering elimination has yet to be fully analysed [70]. Arguably, long-duration infections, which eventually progress to late-stage disease, are captured by the stage 1 exponential distributions used in many modelling frameworks, but modifications could better represent self-cure and nondetection of latent infections in active screening. Many recent modelling studies have concluded that existing vector-control methods have the ability to quickly reduce transmission to and from tsetse to all hosts, and may be critical for elimination in regions where reservoirs exist 67, 68, 69, 70, 71, 72.

**Figure 2:**
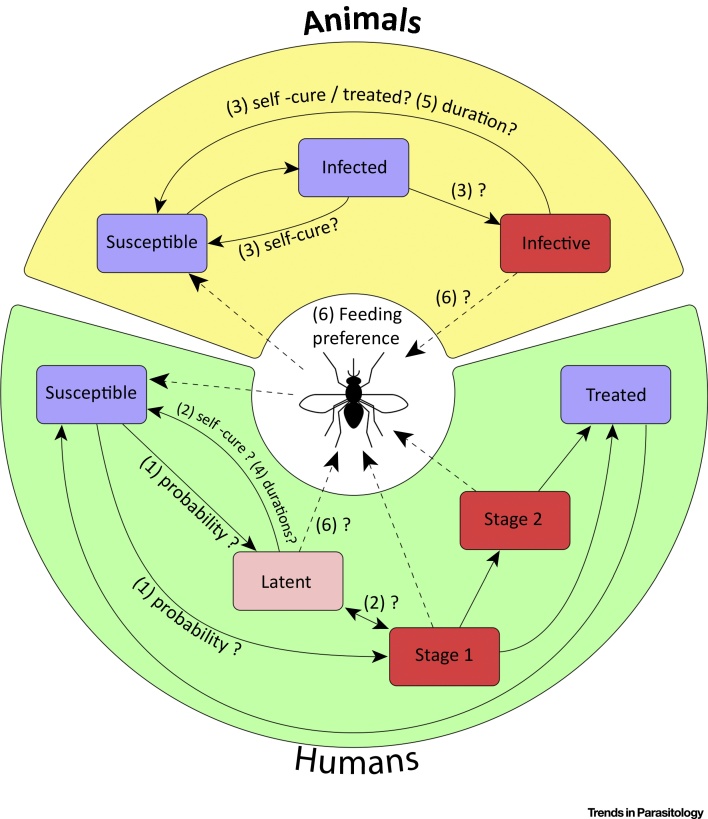
Key Figure: Unknown Elements in Human African Trypanosomiasis Progression and Transmission

New data and investigations into latent human infections and animal infections will help shape the way in which future models are developed and parameterised by factoring in improved biological evidence. Some critical gaps in our knowledge, which influence modelling choices, are shown in Figure 2 (Key Figure). As well as refining formulation and parameterisation of the existing **deterministic** models, it is also clear that a new generation of models is needed. **Stochastic models** are better suited to capture the chance events that determine the role of cryptic reservoirs and their implications for elimination. In conclusion, improved mathematical models on HAT epidemiology, combined with additional field and experimental data, are needed to help understand the respective roles of these reservoirs.

## Concluding Remarks

We believe that attaining the elimination (zero transmission) target of *gambiense*-HAT by 2030 is feasible but, as observed for other neglected tropical diseases, latent infections – whether human or animal – may constitute cryptic parasite reservoirs and thus add another challenge to sustained elimination. To inform evidence-based elimination strategies, a better understanding of the contribution of these putative human and animal reservoirs on the epidemiology of *gambiense-*HAT is required, more in particular on (i) the frequency and duration of latent human infections and infections in animals, (ii) the infectiveness of latent human infections and animal reservoirs to tsetse flies, (iii) the ability of latent human infections or animal reservoirs to sustain transmission in interepidemic periods, and (iv) the possible existence of an animal transmission cycle in the absence of human transmission and its ability to seed a new transmission cycle in humans. To investigate these issues, we urgently need to improve our toolbox for the identification of latent and self-cured infections, including prognostic and diagnostic markers. Also, more accurate and preferably high-throughput tests to detect and monitor *T. b. gambiense* infections in animals should be developed, along with improved mathematical models for exploration of epidemiological hypotheses.

Outstanding Questions How frequent are latent infections with *T. b. gambiense* in humans and in animals?

What is the duration of latent infection in humans and in animals?

How infective are latent human infections and animal reservoirs to tsetse flies?

Are latent human infections or animal reservoirs capable of sustaining transmission in interepidemic periods?

Is it possible to discriminate ongoing latent infection from self-cure in humans?

Do prognostic markers of latent infection outcome in humans exist?

What are the intrinsic and extrinsic factors that influence latent infection outcome in humans?

Can, and do, animal transmission cycles of *T. b. gambiense* exist in the absence of human transmission? If so, what is the likelihood that they could seed a new transmission cycle in humans?

## Glossary

**Anthroponotic disease**: an infectious disease typically transmitted from human to human (including through an insect vector).
**Blood incubation infectivity test**: *T. b. gambiense* and *T. b. rhodesiense* have developed mechanisms to withstand lysis by normal human serum, in contrast to animal infective trypanosomes such as *T. b. brucei*, *T. congolense*, *T. vivax*. To confirm that an animal is infected with *T. b. gambiense* or *T. b. rhodesiense*, its blood, or trypanosomes isolated from that animal, are incubated with human blood or serum whereafter this mixture is injected into a susceptible animal. Only human serum-resistant trypanosomes will be able to initiate an infection in the susceptible animal.
**CATT/*T. b.gambiense***: card agglutination test for trypanosomiasis is an agglutination test for the detection of *gambiense*-specific antibodies in blood. It was the first field-applicable serological test introduced in the 1980s for large-scale screening of populations at risk for *gambiense*-HAT.
**Deterministic mathematical model**: deterministic models ignore the impact of random events, instead capturing average disease dynamics, so that multiple simulations with the same parameter values and initial conditions will lead to exactly the same outcome.
**Elimination of *gambiense*-HAT**: elimination is the reduction to zero of *gambiense*-HAT incidence in a defined area as a result of deliberate efforts; measures to prevent re-emergence are required.
**Elimination of gambiense-HAT as a public health problem**: 90% reduction in areas reporting more than 1 case in 10 000 compared to 2000–2004, and fewer than 2000 annually reported cases globally.
**Eradication of *gambiense*-HAT**: eradication is the permanent reduction to zero of the worldwide incidence of *gambiense*-HAT as a result of deliberate efforts; intervention measures are no longer needed.
**HAT focus**: a geographically defined zone where transmission of HAT occurs or has occurred, to which a geographical name is given (locality, region, and river).
**Immune trypanolysis**: a highly accurate test for *gambiense*-specific antibodies, based on antibody-mediated complement lysis of trypanosomes exposing one single variant-specific antigen on their surface.
**Latent infection**: ongoing infection not progressing to clinical disease; it may remain undiagnosed.
**Maintenance community**: one or more populations which can transmit the pathogen and, together, can maintain the pathogen.
**Maintenance population**: individual populations which can transmit the pathogen and can also maintain the pathogen in the absence of other reservoir populations.
**Rapid diagnostic test (RDT)**: serological antibody- or antigen-detection test, conditioned as individual test, compliant with the ASSURED criteria (Affordable, Sensitive, Specific, User-friendly, Rapid and robust, Equipment-free and Deliverable to end-users); RDTs for *gambiense*-HAT detect antibodies against predominant *gambiense*-specific antigens.
**Reservoir**: a host where the pathogen can maintain itself and from where it can be transmitted to another host; a reservoir host is essential to sustain infection.
**Self-cure**: infection that is cleared by the host without treatment.
**Specificity**: the specificity of a diagnostic test is the probability that the test result is negative when the test person is not infected. It is usually expressed as a percentage and is calculated by dividing the number of test negatives by the number of true negatives x 100.
**Stage determination**: HAT develops from an early stage, with parasites in the peripheral tissues, towards a late stage, with parasite invasion into the central nervous system. Treatment is different for both stages, thus requiring stage determination before drug administration. Determination of the stage is achieved by examination of the cerebrospinal fluid for the presence of trypanosomes and the number of white blood cells.
**Stochastic mathematical model**: stochastic models include chance events so that two simulations with the same parameter values and initial conditions may lead to different outcomes. Chance events become more important at very low prevalences such as in pre-elimination or re-emergent settings.
**Variant surface glycoprotein (VSG)**: in the vertebrate host, the cell surface of trypanosomes is covered with a layer of identical VSGs of one particular variant antigen type (VAT), that protects the trypanosomes against innate immune defence mechanisms of the host; VSGs are highly immunogenic, but periodic switches of the VAT of the VSG coat (antigenic variation) enable the trypanosome to escape the host humoral immune response; during the course of the infection, the host blood contains antibodies against a wide spectrum of different VATs.
**Xenodiagnosis**: diagnostic method based on detection of the parasite in susceptible vectors after they were fed on an individual suspected of being infected with the parasite; in HAT, the vectors used are teneral tsetse flies.

## Resources

^i^www.who.int/gho/neglected_diseases/human_african_trypanosomiasis/en/

^ii^http://apps.who.int/iris/bitstream/10665/70809/1/WHO_HTM_NTD_2012.1_eng.pdf

^iii^www.dndi.org/diseases-projects/hat/portfolio/
