# Supplementary material for: Do Cryptic Reservoirs Threaten Gambiense-Sleeping Sickness Elimination?
Source: Trends Parasitol. 2018 Mar;34(3):197–207. doi: 10.1016/j.pt.2017.11.008 (PMC5840517; doi:10.1016/j.pt.2017.11.008)
Supplement: Supplementary file 1 [file mmc1.pdf]

Trends in Parasitology

Do cryptic reservoirs threaten gambiense-sleeping sickness elimination?

Informal expert group on gambiense HAT reservoirs *

*Correspondence: pbuscher@itg.be (P. Büscher)

Table S1. References and data used to construct the map in Figure 1 on *Trypanosoma brucei gambiense* in non-human mammals.

For the period before 1990, the table below summarises the country and the host species in which T*.b. gambiense* infection has been confirmed by isoenzyme electrophoresis, blood incubation infectivity test or hybridization with DNA probes.

Country	Cattle	Pig	Goat	Sheep	Dog	*Tragelaphus spekii*	*Kobus kob*
Burkina Faso	[S1]						[S2]
Côte d'Ivoire		[S2,S3]			[S1]		
Democratic Republic of the Congo		[S3-S5]	[S5]	[S6]	[S5]		
Equatorial Guinea					[S7]		
Liberia		[S8,S9]			[S3,S8,S10]		
Nigeria	[S11]						
Republic of Congo		[S12]		[S3,S13]			
Uganda						[S14]	

Table S2. References and data used to construct the map in Figure 1 on *Trypanosoma brucei gambiense* in non-human mammals.

For the period after 1990, the table below represents the *T.b. gambiense* positive / total examined animals (and wildlife species) based on *T.b. gambiense* -specific PCR (PCR) and/or immune trypanolysis (TL).

Country	Test	Cattle	Pig	Goat	Sheep	Dog	Wild fauna	Reference
Benin	TL						5/91 belonging to 2/7 species	[S15]
Cameroon	PCR						13/164 belonging to 8/24 species	[S16]
Cameroon	PCR						18/1142 belonging to 8/36 species	[S17]
Cameroon			4/32					[S18]
Cameroon	PCR		1/307	8/264	18/267	0/37		[S19]
Cameroon	PCR		26/225	3/87	10/65	0/20		[S20]
Côte d'Ivoire	TL	7/87	39/137	0/136	0/192			[S21]
Equatorial Guinea	PCR		0/24	3/456	4/218			[S22]
Equatorial Guinea	PCR						15/288 belonging to 11/26	[S23]

							species	
Equatorial Guinea	PCR		0/63	0/21				[S24]
Ghana	PCR	0/146	0/248					[S25]
Guinea	TL	0/158	1/49	1/3	0/103			[S26]
Nigeria	PCR		0/712					[S27]
Uganda	PCR	0/2232	0/161	0/501	0/260	0/113		[S28]
Uganda	PCR	0/203						[S29]

References

S1 Mehlitz, D. et al. (1982) Epidemiological studies on the animal reservoir of Gambiense sleeping sickness. Part III. Characterization of *Trypanozoon* stocks by isoenzymes and sensitivity to human serum. *Tropenmed. Parasitol.* 33, 113-118

S2 Mehlitz, D. (1982) Trypanosomes in African wild mammals. In *Perspectives in Trypanosomiasis Research* (Baker,J.R., ed), pp. 25-35, Wiley Research Studies Press

S3 Paindavoine, P. et al. (1986) The use of DNA hybridization and numerical taxonomy in determining relationships between *Trypanosoma brucei* stocks and subspecies. *Parasitology* 92, 31-50

S4 Kageruka, P. et al. (1977) Strain of *Trypanosoma* (*Trypanozoon*) *brucei* isolated from pigs in Bas-Zaire. *Ann Soc Belg Med Trop* 57, 85-88

S5 Van Hoof, L.M.J.J. (1947) Observations on trypanosomiasis in the Belgian Congo. *Trans. R. Soc. Trop. Med. Hyg.* 40, 728-761

S6 Truc, P. and Tibayrenc, M. (1993) Population genetics of Trypanosoma brucei in central Africa: taxonomic and epidemiological significance. *Parasitology* 106 (Pt 2), 137-149

S7 Denecke, K. (1941) Menschenpathologene Trypanosomes des Hundes auf Fernando Poo. Ein Beitrag zur Epidemiologie des Schlafkrankheit. *Archiv für Hygiene und Bakteriologie* 126, 38-42

S8 Gibson, W. et al. (1978) The identification of *Trypanosoma brucei gambiense* in Liberian pigs and dogs by isoenzymes and by resistance to human plasma. *Tropenmed. Parasitol.* 29, 335-345

S9 Mehlitz, D. (1977) The behaviour in the blood incubation infectivity test of four *Trypanozoon* strains isolated from pigs in Liberia. *Trans. R. Soc Trop Med Hyg.* 71, 86

S10 Zillmann, U. et al. (1984) Identity of trypanosome stocks isolated from man -and a domestic dog in Liberia. *Trop. Geogr. Med.* 35, 105-108

S11 Joshua, R.A. et al. (1983) Isolation of human serum resistant *Trypanozoon* from cattle in Nigeria. *Tropenmed. Parasitol.* 34, 201-202

S12 Truc, P. et al. (1991) Multilocus isozyme identification of *Trypanosoma brucei* stocks isolated in Central Africa: evidence for an animal reservoir of sleeping sickness in Congo. *Acta Trop.* 49, 127-135

S13 Scott, C.M. et al. (1983) The sheep as a potential reservoir of human trypanosomiasis in the Republic of the Congo. *Trans. R. Soc. Trop. Med. Hyg.* 77, 397-401

S14 Duke, H.L. (1921) On the zoological status of the polymorphic mammalian trypanosomes of Africa and their relation to man. *Parasitology* 13, 351-397

S15 Guedegbe, B. et al. (1992) Indications sérologiques de l'existence d'un réservoir sauvage du *Trypanosoma brucei gambiense* dans la réserve de la biosphère de la Pendjari en République du Bénin. *Ann. Soc. Belg. Méd. Trop.* 72, 113-120

S16 Herder, S. et al. (2002) Identification of trypanosomes in wild animals from Southern Cameroon using the polymerase chain reaction (PCR). *Parasite* 9, 345-349

S17 Njiokou, F. et al. (2006) Wild fauna as a probable animal reservoir for *Trypanosoma brucei gambiense* in Cameroon. *Infect Genet. Evol.* 6, 147-153

S18 Nkinin, S.W. et al. (2002) Characterization of *Trypanosoma brucei s.l.* subspecies by isoenzymes in domestic pigs from the Fontem sleeping sickness focus of Cameroon. *Acta Trop.* 81, 225-232

S19 Njiokou, F. et al. (2010) Domestic animals as potential reservoir hosts of *Trypanosoma brucei gambiense* in sleeping sickness foci in Cameroon. *Parasite* 17, 61-66

S20 Njitchouang, G.R. et al. (2010) Analysis of the domestic animal reservoir at a microgeographical scale, the Fontem sleeping sickness focus (South-West Cameroon). *Journal of Cell and Animal Biology* 4, 73-80

S21 N'Djetchi, M.K. et al. (2017) The study of trypanosome species circulating in domestic animals in two human African trypanosomiasis foci in Côte d'Ivoire identifies pigs and cattle as potential reservoirs of *Trypanosoma brucei gambiense*. *PLoS Negl. Trop Di* 11, e0005993

S22 Cordon-Obras, C. et al. (2009) *Trypanosoma brucei gambiense* in domestic livestock of Kogo and Mbini foci (Equatorial Guinea). *Trop. Med. Int. Health* 14, 535-541

S23 Cordon-Obras, C. et al. (2015) Molecular evidence of a *Trypanosoma brucei gambiense* sylvatic cycle in the human african trypanosomiasis foci of Equatorial Guinea. *Front Microbiol.* 6, 765

S24 Cordon-Obras, C. et al. (2010) Screening of *Trypanosoma brucei gambiense* in domestic livestock and tsetse flies from an insular endemic focus (Luba, Equatorial Guinea). *PLoS Negl. Trop. Dis.* 4, e704

S25 Nakayima, J. et al. (2012) Molecular epidemiological studies on animal trypanosomiases in Ghana. *Parasit. Vectors* 5, e217

S26 Kagbadouno, M.S. et al. (2012) Epidemiology of sleeping sickness in Boffa (Guinea): where are the trypanosomes? *PLoS Negl. Trop. Dis.* 6, e1949

S27 Karshima, S.N. et al. (2016) Eco-epidemiology of porcine trypanosomosis in Karim Lamido, Nigeria: prevalence, seasonal distribution, tsetse density and infection rates. *Parasit. Vectors* 9, 448

S28 Balyeidhusa, A.S. et al. (2012) Apparent lack of a domestic animal reservoir in *gambiense* sleeping sickness in northwest Uganda. *Vet. Parasitol.* 187, 157-167

S29 Jing, Z. et al. (2009) A field study to estimate the prevalence of bovine African trypanosomosis in Butaleja District, Uganda. *J. Vet. Med Sci.* 71, 525-527
